# Influence of Short and Long Hyperglycemia on Cardioprotection by Remote Ischemic Preconditioning—A Translational Approach

**DOI:** 10.3390/ijms232314557

**Published:** 2022-11-22

**Authors:** Katharina Feige, Sebastian Roth, René M’Pembele, Anna Galow, Sarah Koenig, Martin Stroethoff, Annika Raupach, Giovanna Lurati Buse, Alexander M. Mathes, Markus W. Hollmann, Ragnar Huhn, Carolin Torregroza

**Affiliations:** 1 Department of Anesthesiology, Medical Faculty and University Hospital Duesseldorf, Heinrich-Heine-University Duesseldorf, Moorenstr. 5, 40225 Duesseldorf, Germany; 2 Department of Anesthesiology and Intensive Care Medicine, Faculty of Medicine and University Hospital of Cologne, University of Cologne, Kerpener Str. 62, 50937 Cologne, Germany; 3 Department of Anesthesiology, Amsterdam University Medical Center (AUMC), Location AMC, Meiberdreef 9, 1105 AZ Amsterdam, The Netherlands; 4 Department of Anesthesiology, Kerckhoff-Clinic GmbH, Heart & Lung Center, Benekestr. 2-8, 61231 Bad Nauheim, Germany

**Keywords:** remote ischemic preconditioning, cardioprotection, reperfusion injury, hyperglycemia

## Abstract

The adverse impact of common diseases like diabetes mellitus and acute hyperglycemia on morbidity and mortality from myocardial infarction (MI) has been well documented over the past years of research. In the clinical setting, the relationship between blood glucose and mortality appears linear, with amplifying risk associated with increasing blood glucose levels. Further, this seems to be independent of a diagnosis of diabetes. In the experimental setting, various comorbidities seem to impact ischemic and pharmacological conditioning strategies, protecting the heart against ischemia and reperfusion injury. In this translational experimental approach from bedside to bench, we set out to determine whether acute and/or prolonged hyperglycemia have an influence on the protective effect of transferred human RIPC-plasma and, therefore, might obstruct translation into the clinical setting. Control and RIPC plasma of young healthy men were transferred to isolated hearts of young male Wistar rats in vitro. Plasma was administered before global ischemia under either short hyperglycemic (HGs Con, HGs RIPC) conditions, prolonged hyperglycemia (HGl Con, HGl RIPC), or under normoglycemia (Con, RIPC). Infarct sizes were determined by TTC staining. Control hearts showed an infarct size of 55 ± 7%. Preconditioning with transferred RIPC plasma under normoglycemia significantly reduced infarct size to 25 ± 4% (*p* < 0.05 vs. Con). Under acute hyperglycemia, control hearts showed an infarct size of 63 ± 5%. Applying RIPC plasma under short hyperglycemic conditions led to a significant infarct size reduction of 41 ± 4% (*p* < 0.05 vs. HGs Con). However, the cardioprotective effect of RIPC plasma under normoglycemia was significantly stronger compared with acute hyperglycemic conditions (RIPC vs. HGs RIPC; *p* < 0.05). Prolonged hyperglycemia (HGl RIPC) completely abolished the cardioprotective effect of RIPC plasma (infarct size 60 ± 7%; *p* < 0.05 vs. HGl Con; HGl Con 59 ± 5%).

## 1. Introduction

The adverse impact of common diseases like diabetes mellitus and acute hyperglycemia on morbidity and mortality from myocardial infarction (MI) has been well defined over the past years of research.

Hyperglycemia is commonly documented during the perioperative period in patients undergoing cardiac surgery [1]. Interestingly, not only chronic diabetes mellitus but also episodes of acute hyperglycemia seem to be an independent, outcome-related risk factor for patients suffering from acute myocardial infarction, irrespective of pre-existing diabetes mellitus [2,3]. Clinical evidence has shown that acute hyperglycemia is independently associated with larger myocardial infarct size and further with impaired left ventricular function in both diabetic and nondiabetic patients [4,5,6].

Until now, causal therapy in the form of immediate restoration of coronary blood circulation, either as a percutaneous coronary intervention or as a coronary bypass surgery, remains the gold standard for patients suffering from MI [7]. Paradoxically, resupply of coronary blood flow itself provokes ischemia-reperfusion injury (I/R), restraining the outcome of the reperfusion-intervention. Based on this phenomena, cardioprotective approaches reducing the negative side effects of unpredictable I/R injury are of great clinical relevance. One of these protective mechanisms is remote ischemic preconditioning (RIPC)—described as short cycles of transient ischemia and reperfusion of peripheral tissue (e.g., upper limb), increasing the tolerance of the myocardial tissue against a subsequent I/R injury [8]. It is known that RIPC stimuli lead to the release of protective humoral factors involved in transmitting cardioprotection in the target organ [9]. Although experimental data on RIPC in almost all animal species have been a promising approach to induce cardioprotection before a subsequent index ischemia, translation into the clinical setting still remains difficult [10]. The two large, well-known clinical randomized trials, ERICCA [11] and RIPHeart [12], failed to show an improvement in clinical outcome from patients treated with RIPC undergoing cardiac surgery. Increasing concerns arise regarding the effect of RIPC in humans in the context of confounders such as age, sex, and comorbidities. Multiple studies [13,14,15] have shown that various comorbidities and/or comedication have an influence on cardioprotective properties; in particular, acute hyperglycemia, which is frequently observed in patients with cardiovascular diseases, perchance as an acute stress response. In the clinical setting, it is discussed whether hyperglycemia directly contributes to the adverse outcome or whether it is more a predictor of higher risk. Nevertheless, the relationship between blood glucose and mortality appears linear, independently of a diagnosis of diabetes [3].

Based on these findings, in this study, we focused on investigating a possible effect of isolated acute and prolonged hyperglycemia on RIPC. For this translational approach from bedside to bench, we employed RIPC plasma collected from young, healthy, male volunteers. Plasma was transferred to an isolated rat heart under either temporary acute hyperglycemic conditions or prolonged hyperglycemia, without distortion by other confounders. We set out to determine whether acute and/or prolonged hyperglycemia has an influence on the protective effect of transferred human RIPC plasma and, therefore, might obstruct translation into the clinical setting.

## 2. Results

### 2.1. Animal Characteristics

No differences were detected between and within all groups of this study regarding body weight, wet heart weight, and level or time of maximal ischemic contracture (Table 1).

### 2.2. Infarct Size Measurement

**Figure 1:**
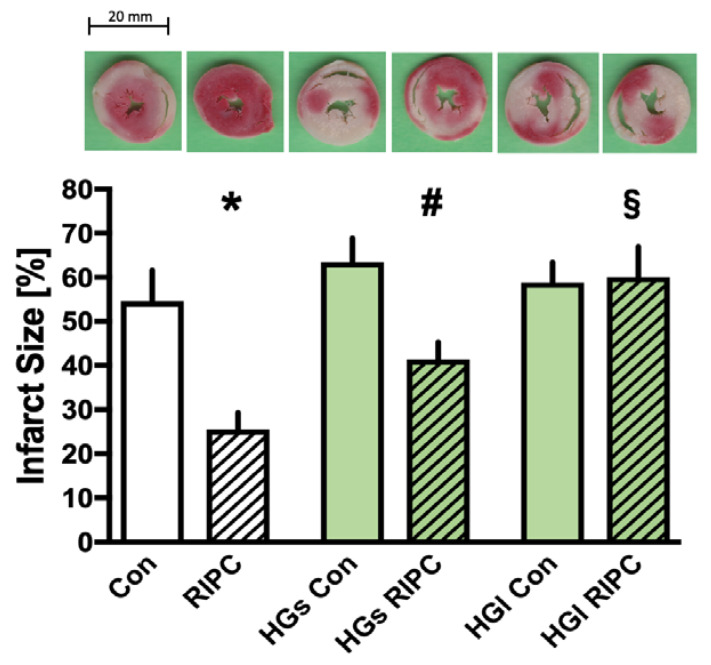
Infarct size measurement. Histogram shows all infarct sizes of the study. Data are presented as means ± SD, * *p* < 0.05 vs. Con, # *p* < 0.05 vs. HGs Con, § *p* < 0.05 vs. RIPC.

All infarct sizes are demonstrated in Figure 1. Control hearts showed an infarct size of 55 ± 7% of the left ventricle. Preconditioning with transferred RIPC plasma from young, healthy men significantly reduced infarct size to 25 ± 4% (RIPC, *p* < 0.05 vs. Con). Under short hyperglycemia, control hearts (HGs Con) showed an infarct size of 63 ± 5%. Applying RIPC plasma under short hyperglycemic conditions (HGs RIPC) leads to a significant infarct-size-reducing effect (HGs RIPC 41 ± 4%; *p* < 0.05 vs. HGs Con). Under long hyperglycemic conditions, infarct size measured 59 ± 5% in control hearts (HGl Con). Preconditioning with RIPC plasma under prolonged hyperglycemia (HGl RIPC) did not induce infarct size reduction compared with the control (HGl RIPC 60 ± 7%; vs. HGl Con). Notably, the protective effect of RIPC plasma under short hyperglycemia (HGs RIPC) is considerably attenuated compared with RIPC, but still significantly reduces infarct size (HGs RIPC 41 ± 4%; *p* < 0.05 vs. RIPC). In contrast, long hyperglycemia completely abolishes the positive impact of preconditioning with RIPC plasma, compared with normoglycemia and short hyperglycemia (HGl RIPC 60 ± 7%; *p* < 0.05 vs. RIPC and HGs RIPC).

### 2.3. Cardiac Function and Glucose Values

Hemodynamic data from all groups are pictured in Table 2. As shown, no differences were observed for heart rate between all groups. In all groups, LVDP and coronary flow significantly decreased during the reperfusion period compared with baseline. Appling glucose solution before ischemia significantly elevated glucose levels in HGs Con and HGs RIPC compared with Con, normalizing at the beginning of reperfusion (not statistically different compared with Con). For the subgroups HGl Con and HGl RIPC, significantly elevated glucose levels can be documented during preconditioning and reperfusion compared with baseline.

## 3. Discussion

In the present study, we focused on the influence of disturbed glucose hemostasis on cardioprotection induced by transferred human RIPC plasma.

Our results demonstrate that prolonged hyperglycemia fully abolishes the beneficial effect of transferred RIPC plasma on infarct size after I/R injury. Interestingly, a short transient episode of hyperglycemia before ischemia—during preconditioning with transferred RIPC plasma—does not completely abrogate cardioprotection. RIPC plasma transfer in fact still induces infarct size reduction; however, it is less pronounced compared with under normoglycemic conditions.

### 3.1. RIPC Plasma Transfer under Normoglycemia

Owing to its noninvasive nature and high practicability, RIPC maneuver seems to be an optimal therapeutic strategy inducing inter-organ protection against I/R injury. Multiple pieces of experimental evidence [10,16,17] exist supporting the beneficial effects of RIPC, as it enhances the ability of the heart to sustain prolonged I/R insults. Keeping in mind, most experimental studies implement I/R injury in the absence of cardiovascular risk factors—using an animal model. Transferred clinical studies on RIPC in humans did not conclusively verify these beneficial effects of RIPC. The two large, well-known clinical trials, ERRICA [11] and RIPHeart [12], showed neutral results with regards to their chosen composite primary endpoint (e.g., cardiovascular death, myocardial infarction, coronary revascularization, stroke after 12 months) and for the level of troponin T release in patients undergoing cardiac surgery.

These discrepancies between experimental evidence and clinical ineffectiveness are attributed to confounding variables such as choice of anesthetic regime, concomitant medication, patient’s characteristic, or comorbidities like diabetes and hyperglycemia [18].

In our study, we performed an inter-species transfer of human RIPC plasma on an isolated rat heart under both normal and disturbed glucose hemostasis. The results demonstrate a powerful cardioprotective effect of transferred human RIPC plasma significantly reducing infarct size from 55 ± 7% of the left ventricle to 25 ± 4% under normoglycemic conditions. Previously, Heinen et al. [19] investigated whether humoral factors in RIPC plasma of young male volunteers activate common cardioprotective signaling pathways in a healthy rat heart. They showed that the respective RIPC plasma increased phosphorylation of glycogen synthase kinase 3ß (GSK3ß), as a downstream target of the reperfusion injury salvage kinase (RISK) pathway. The increase in phosphorylation leads to the inhibition of GSK3ß, then preventing the opening of the mitochondrial permeability transition pore (mPTP), a key player in cardioprotection. Interestingly, in contrast to plasma from young male volunteers, GSK3ß phosphorylation was not altered after application of RIPC plasma from old men.

The viability of transferring human blood samples after RIPC to an isolated heart, initiating cardioprotection, supports the concept of the release of humoral factors as an important signaling step of the inter-organ communication [20]. Although the exact molecular structure and details of the release mechanism remain partly unknown, Hildebrandt et al. suggest that these factors are hydrophobic, thermolabile low-molecular mass molecules (>3.5; <15 kDa), circulating up to 6 days in the blood stream [21,22,23]. Heusch et al. [10] discuss the role of cytokines and chemokines (interleukin-10, stromal cell-derived factor-1α, substance P), glucagon-like peptide-1 (GLP-1), and NOS as possible humoral factors.

Our result, showing that transferred RIPC plasma from young male volunteers has a strong infarct-limiting effect, is in line with previous findings, indicating that humoral factors can be transferred to a naïve heart to initiate cardioprotection.

### 3.2. Influence of Short and Prolonged Hyperglycemia on Transferred RIPC Plasma

Different clinical studies show evidence that the concomitant occurrence of hyperglycemia in patients suffering myocardial infarction increases the risk of mortality and morbidity, irrespective of whether the patient is diagnosed with diabetes [2,24]. Interestingly, hyperglycemia is known to interact with different ischemic conditioning strategies, e.g., ischemic preconditioning and remote ischemic preconditioning (RIPC), as well as pharmacological strategies [25,26,27] in experimental studies. This interference of hyperglycemia might be one relevant confounder obstructing the implementation of promising experimental cardioprotective strategies into the clinical routine.

The detailed mechanism of hyperglycemia blocking cardioprotection is yet not fully revealed, but has been one major focus of cardioprotective research, acquiring increasing knowledge. In this context, Craig et al. [28] described an elevation in the ATP level under hyperglycemia, which inhibits the activation of cellular ATP-sensitive potassium (K_ATP_) channels. Similar K_ATP_ channels are located in the mitochondrial membrane and have been proven to play a key role in different cardioprotective strategies. Their activation ultimately results in suppression of mPTP opening, inducing myocardial protection [29,30]. In the context of RIPC, Kristiansen et al. [31] discussed the role of mK_ATP_ channels, showing that a nonselective blockade of mK_ATP_ channels completely eliminated the cardioprotective effect of RIPC in a donor heart. Especially during reperfusion, the activation of mK_ATP_ channels plays a crucial role in cascades inducing cardioprotection. Thus, it might be considerable that, during prolonged hyperglycemia, the effect of excessively, ongoing elevated ATP levels on mK_ATP_-channels—especially in the reperfusion—is more pronounced compared with the subgroup of short hyperglycemia. Ergo, the activation of mK_ATP_ channels might be only partly affected under short episode of hyperglycemia, still initiating cardioprotective signaling cascades.

Besides the changes in ATP level, hyperglycemia induces an excessive mitochondrial overproduction of ROS, causing matrix swelling, release of pro-apoptotic factors, opening of mPTP, and finally cell death [32,33,34].

Lastly, acute hyperglycemia seems to block different parts of myocardial signaling cascades involved in the cardioprotective properties of RIPC, such as Akt phosphorylation, endothelial NO synthase (eNOS), or protein kinase G (PKG) [35,36].

Interestingly, the results from our study report that transferred RIPC plasma under short, transient hyperglycemia still induces a cardioprotective effect, even though it is less profound compared with the infarct size reduction under normoglycemia. In contrast, prolonged hyperglycemia completely abolished myocardial protection by RIPC plasma.

This distinct difference in results indicates that the duration and/or timing of hyperglycemia might be an important factor in the context of blocked cardioprotection.

As the results demonstrate, peracute hyperglycemia before ischemia only partly interferes with RIPC-induced preconditioning, while prolonged hyperglycemic conditions during reperfusion seem to be critical. In line with our data are the findings of Kehl et al. [35,37], showing that severe hyperglycemia blocks isoflurane-induced preconditioning, whereas moderate hyperglycemic conditions only party interact with this conditioning strategy. They concluded that hyperglycemia attenuates or completely abolishes the protective effects of isoflurane in a dose-related manner caused by an excessive generation of ROS. While the release of small amounts of ROS, e.g., owing to opening of mK_ATP_ channels, is involved in cardioprotection, excessive amounts of ROS lead to opening of the mPTP, which ultimately induces cell damage [38,39,40]. Hence, next to ATP levels, the amount of produced ROS seems to be an important factor in the influence of hyperglycemia on cardioprotection by RIPC.

In line with our findings, Wider et al. [41] report that serum harvested from normoglycemic rats subjected to an RIPC stimulus confers protection when transferred to HL-1 cardiomyocytes exposed to ischemia-reperfusion. However, the transfer of serum from hyperglycemic Zucker fatty rats, a model of type-2 diabetes with modest elevation in blood glucose, failed to limit cardiomyocyte death. Torregroza et al. [42] demonstrated similar results investigating animal plasma transfer, in a similar experimental setting as our current study. Plasma sampled from healthy young rats after an RIPC stimulus in vivo transferred onto isolated rat hearts in vitro induced a significant infarct size reduction. In contrast, under hyperglycemia, the protective effects of RIPC plasma transfer were completely abolished.

Clinical studies indicate that myocardium of patients suffering from diabetes mellitus may be susceptible to episodes of ischemia/reperfusion, per se causing larger infarct sizes [43]. Further, even a brief exposure of the myocardium to hyperglycemic conditions has been shown to abolish the protective effect of ischemic conditioning, implying that hyperglycemia itself may be responsible for the resistance to cardioprotection associated with diabetes [25,44,45]. However, the results from the above-mentioned experimental protocol by Wider et al. did not support these clinical implications. This discrepency between experimental and clinical studies underlines the need for more profound research on potential mechanistics on this topic.

However, not only the condition of the myocardium as a target itself seems to be relevant for conditioning strategies with RIPC plasma, but also patients’ comorbidities influencing the release of mediating humoral factors. Jensen et al. [46] described the phenonema of reduced infarct size and hemodynamic recovery after preconditioning with RIPC plasma of both non-diabetic and diabetic patients without peripheral neuropathy. However, in the subgroup of diabetic patients with neuropathy, the cardioprotective effect was attenuated. They conclude that the release mechanism involves neural pathways being inefficient in patients suffering long-term effects of diabetes mellitus. Besides the affected release mechanisms of humoral factors, several confounding factors have been investigated in the context of cardioprotection and are indicated to block conditioning strategies in the clinical setting [47]. In our previous study [48], we have investigated the influence of diabetes mellitus on the release of humoral factors after RIPC in patients undergoing coronary artery bypass graft (CABG) surgery. We employed a comparable experimental ischemia- and reperfusion setup using human plasma transferred onto isolated rat hearts in vitro. In contrast to our current study, plasma was taken from middle-aged patients receiving propofol-free anesthesia before undergoing CABG surgery. Data showed that plasma of these respective patients lacked any cardioprotective effect. Interestingly, these findings were completely independent of a pre-existing diabetes mellitus.

While most of the confounders are difficult to alter (comorbidities like hypertension or hypertrophy, comedications) or cannot be changed at all (age or gender), hyperglycemia is fairly easy to modify, especially in the perioperative setting. Therefore, the results from our current study are of critical importance and emphasize the significant role of preserving or establishing normoglycemia with regard to myocardial conditioning. Nevertheless, further research is needed investigating the various patient characteristics. The translational concept, focusing not only on bench to bedside, but also bedside to bench, might be one promising approach in this context.

There are limitations of our study that need to be mentioned. The induction of short and prolonged hyperglycemia only simulates a temporary state of physiological conditions rather than the complex pathophysiology of a chronic diabetes mellitus disease. As a first step, this current study was designed to focus on one main comorbidity, deliberately, the fact that patients with hyperglycemia or even diabetes mellitus often suffer from multiple cardiovascular risk factors. A model representing the chronic stages of the disease should be a follow-up-investigation. In this study, we address the question of whether RIPC plasma mediates cardioprotection in the presence of hyperglycemia, employing a translational approach with an inter-species plasma transfer using human RIPC plasma. We acknowledge that more extensive research on the exact underlying mechanisms is required for generating specific clinical interventions that are potent for protecting the heart against ischemia-reperfusion injury. Considering the above-mentioned research, future studies should focus on mitochondrial ATP levels and mK_ATP_ channels in the context of blocked cardioprotection under hyperglycemia.

## 4. Materials and Methods

Investigations were approved by the local ethics committee (#2018-160_1) and the local Animal Care and Use Committee of the University of Duesseldorf (project number O27/12). All experiments included in this study were conducted in accordance with the Guide for the Care and Use of Laboratory Animals published by the U.S. National Institute of Health (NIH publication No. 85-23, revised 1996). The results were reported according to the ARRIVE guidelines.

### 4.1. Part 1: RIPC-Plasma Collection in Volunteers

**Figure 2:**
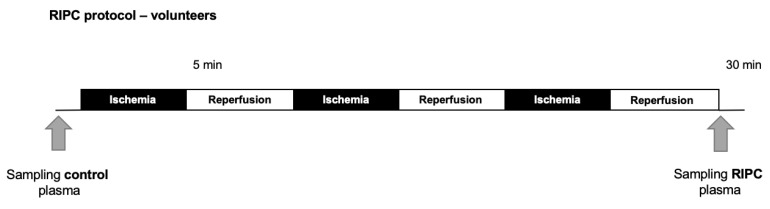
Schematic diagram of the experimental protocol (Part 1) for induction of control (Con) and RIPC blood plasma sampled in young, male volunteers. RIPC was induced by three cycles of ischemia (5 min) at the upper left arm, followed by 5 min of reperfusion.

After written informed consent, plasma was collected from 10 healthy, young (age 18–30 years), normal body weight (body mass index; BMI: 24.6 ± 3.4 kg/m^2^) male adult volunteers undergoing a protocol of RIPC-maneuver. Demographic data and criteria of inclusion/exclusion of the probands are shown in Table 3 and Table 4. The RIPC protocol consisted of three cycles (5 min each) of upper arm ischemia alternating with 5 min of reperfusion (Figure 2). Ischemia was induced by inflating a standard blood pressure cuff at the left forearm to a pressure of 200 mmHg, verifiable exceeding the systolic blood pressure. Reperfusion started by deflating the cuff. After performing the described maneuver of ischemia and reperfusion, 50 mL venous blood samples were collected from the cubital vein of the opposite arm immediately after the last cycle of reperfusion. A control (Con) blood sample was taken prior to the first cycle of ischemia, respectively. All blood samples were collected in EDTA-monovettes, then centrifuged at 3000 rpm for 10 min at 10 °C. The extracted plasma was stored at −80 °C until transfer to the isolated rat heart.

### 4.2. Part 2: (a) Experimental Setting—Langendorff Isolated Heart

All in vitro experiments were performed in the isolated male rat heart. The surgical preparation was performed as described in detail previously [49]. Male Wistar rats (2–3-month-old) were anesthetized with intraperitoneal injection of pentobarbital (80 mg/kg body weight, Narcoren, Merial, Germany) and then decapitated. Hearts were excised via a median thoracotomy, immediately mounted onto a Langendorff-System, and perfused with Krebs–Henseleit–Buffer (KHB; containing 118 mM NaCl, 4.7 mM KCl, 1.2 mM MgSO_4_, 1.17 mM KH_2_PO_4_, 24.9 mM NaHCO_3_, 2.52 mM CaCl_2_, 11 mM glucose, and 1 mM lactate) under constant pressure (80 mmHg) at a temperature of 37 °C. The Krebs–Henseleit buffer was supplemented with a mixture of 95% O_2_ and 5% CO_2_. A fluid-filled balloon was inserted into the left ventricle, measuring standardized hemodynamic parameters, setting the baseline left ventricular end-diastolic pressure to 4–6 mmHg. For all experiments, heart rate, left ventricular end-systolic pressure (LVESP), and left ventricular end-diastolic pressure (LVEDP) were continuously measured and digitized by an analogue to digital converter (PowerLab/8SP, ADInstruments Pty Ltd., Castle Hill, Australia) at a sampling rate of 500 Hz. Data were recorded using Labchart 8.0 for Windows (ADInstruments Pty Ltd., Castle Hill, Australia). Left ventricular developed pressure (LVDP) was calculated as LVESP–LVEDP.

Coronary flow (CF) and glucose level in the coronary effluent were measured at given time points (see Table 2). Additionally, maximal contracture during ischemia and the respective time-point was analyzed for each experiment as an indicator of cardiomyocyte damage.

After successfully completing the experiment at the end of reperfusion, each heart was removed from the Langendorff system and cut into eight transverse slices (2 mm each slice) to examine the infarct size. A 0.75% triphenyltetrazoliumchloride (TTC) solution was applied to investigate the infarcted area compared with viable tissue. A blinded, experienced investigator analyzed infarct sizes using planimetry (SigmaScan Pro5 software), determined as the percentage of infarct area per total area of the left ventricle [50].

### 4.3. Part 2: (b) Experimental Protocol

**Figure 3:**
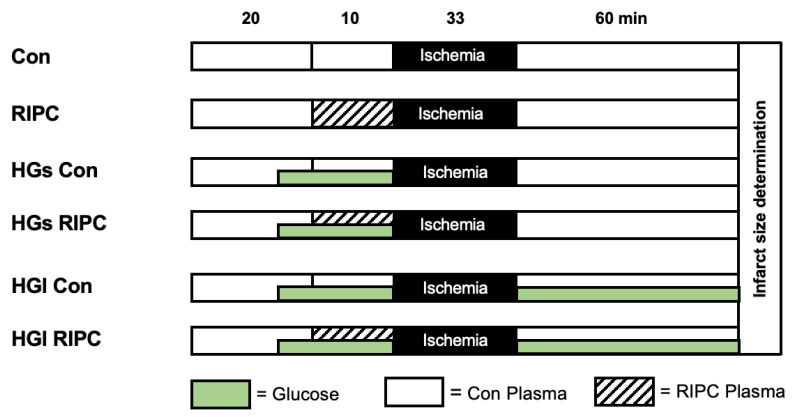
Experimental protocol (Part 2). **Con** = control plasma; **RIPC** = RIPC plasma; **HG** = hyperglycemia; **HGs** = hyperglycemia short; **HGl** = hyperglycemia long; **Green bar** = hearts were perfused with a total of 22 mmol/L glucose concentration. **Control** (**Con**)**:** hearts were perfused with control plasma for 10 min prior ischemia under normoglycemia, short (**HGs Con**), or long (**HGl Con**) hyperglycemia, respectively. **RIPC:** hearts were perfused with RIPC plasma for 10 min prior ischemia under normoglycemia, short (**HGs RIPC**), or long (**HGl RIPC**) hyperglycemia respectively.

Hearts were randomized into six experimental groups (*n* = 4–5 per group), as shown in Figure 3.

All hearts underwent 20 min of equilibration period, 10 min of preconditioning period, 33 min of ischemia, followed by 60 min of reperfusion. Global ischemia was induced by complete suppression of retrograde perfusion via the Langendorff Apparatus. At the end of ischemia, perfusion was restored to initiate the reperfusion period. Prior to ischemia, hearts were perfused for 10 min as a preconditioning intervention with either control (Con) or RIPC (RIPC) plasma from volunteers. Preconditioning with human RIPC plasma was achieved by administrating undiluted plasma via a syringe pump (Perfusor Space, B.Braun, Melsungen, Germany) at an infusion rate of 1% of the coronary flow. Plasma was not dissolved but applied purely.

To induce hyperglycemia, hearts were perfused with a total concentration of 22 mmol/L glucose (a 11 mmol/L glucose solution in addition to KHB already containing 11 mmol/L glucose). Glucose treatment was started 2 min prior to perfusion with Con or RIPC plasma to ensure hyperglycemic conditions at the start of preconditioning. The protocol for induction of hyperglycemia was taken from our previous investigations [15]. Hyperglycemic conditions were maintained for either a short period until the start of ischemia (HGs) or prolonged until the end of reperfusion (HGl). Control plasma (HGs Con/HGl Con) and RIPC plasma (HGs RIPC/HGl RIPC) were administered as a preconditioning intervention in the respective groups under either short or long hyperglycemic conditions. All substances were applied at an infusion rate of 1% of coronary flow.

**Con:** Hearts were perfused with control plasma for 10 min as preconditioning under normoglycemia.

**RIPC**: Hearts were perfused with RIPC plasma for 10 min as preconditioning under normoglycemia.

**HGs Con:** Hearts were perfused with control plasma for 10 min as preconditioning under short hyperglycemia.

**HGs RIPC:** Hearts were perfused with RIPC plasma for 10 min as preconditioning under short hyperglycemia.

**HGl Con:** Hearts were perfused with control plasma for 10 min as preconditioning under long hyperglycemia.

**HGl RIPC:** Hearts were perfused with RIPC plasma for 10 min as preconditioning under long hyperglycemia.

### 4.4. Statistical Analysis

Sample size calculation (GraphPad StatMate™, GraphPad Software, San Diego, CA, USA) revealed a group size of *n* = 4 for detecting a 25% mean difference and a standard deviation of 15% in infarct size (power 80%, α < 0.05 (two-tailed)). We performed a two-way analysis of variance (ANOVA) and a Tukey post hoc test (GraphPad Software V7.01, San Diego, CA, USA) for comparison of hemodynamic data and glucose levels between groups as well as between different time points within groups. Data are presented as mean ± standard deviation (SD). Infarct sizes were analyzed by a one-way ANOVA and a Tukey´s post hoc test. *p* < 0.05 was considered statistically significant for changes within and between groups.

## 5. Conclusions

With this bed to benchside approach, we demonstrate that cardioprotection can be induced by transfer of human RIPC plasma to the isolated rat heart. The simultaneous occurrence of hyperglycemia interferes with the cardioprotective effect of RIPC. Interestingly, this blockage depends on the duration of hyperglycemia. While prolonged hyperglycemia completely blocks infarct size reduction by RIPC plasma, myocardial protection by transfer of RIPC plasma under short transient hyperglycemia—although less pronounced—is still effective.

## Figures and Tables

**Table 1:** Weights and ischemic contracture.

	*n*	Body Weight (g)	Heart Weight Wet (g)	Time of Max. Ischemic Contracture (min)	Level of Max. Ischemic Contracture (mmHg)
Con	4	325 ± 9	1.17 ± 0.09	20 ± 2	62 ± 16
RIPC	4	293 ± 16	1.05 ± 0.03	22 ± 3	60 ± 9
HGs Con	5	312 ± 21	1.16 ± 0.04	19 ± 2	75 ± 8
HGs RIPC	4	312 ± 21	1.16 ± 0.04	19 ± 2	75 ± 8
HGl Con	4	309 ± 19	1.10 ± 0.03	19 ± 3	79 ± 10
HGl RIPC	4	297 ± 28	1.07 ± 0.02	19 ± 1	67 ± 22

Data are mean ± SD; Con = control; RIPC = temote ischemic preconditioning; HGs = hyperglycemia short; HGl = hyperglycemia long.

**Table 2:** Hemodynamic variables and glucose values.

	Baseline	PC	Reperfusion	
			30	60
Heart Rate (bpm)				
Con	277 ± 13	259 ± 25	246 ± 80	218 ± 69
RIPC	312 ± 43	295 ± 46	237 ± 94	225 ± 91
HGs Con	305 ± 32	300 ± 30	309 ± 55	238 ± 57
HGs RIPC	304 ± 36	282 ± 26	290 ± 42	263 ± 81
HGl Con	282 ± 11	279 ± 9	235 ± 28	236 ± 29
HGl RIPC	266 ± 5	269 ± 6	273 ± 20	226 ± 42
Left Ventricular Developed Pressure (mmHg)				
Con	130 ± 12	134 ± 10	15 ± 11 *	25 ± 16 *
RIPC	130 ± 4	145 ± 19	51 ± 19 *	51 ± 7 *
HGs Con	137 ± 8	139 ± 12	28 ± 26 *	28 ± 12 *
HGs RIPC	125 ± 14	125 ± 11	24 ± 8 *	30 ± 10 *
HGl Con	131 ± 17	125 ± 18	19 ± 14 *	15 ± 10 *
HGl RIPC	141 ± 3	138 ± 5	10 ± 8 *	27 ± 12 *
Left Ventricular End-Diastolic Pressure (mmHg)				
Con	5 ± 1	5 ± 1	86 ± 8 *	76 ± 7 *
RIPC	4 ± 1	3 ± 1	74 ± 4 *^§^	71 ± 7 *
HGs Con	5 ± 1	5 ± 1	81 ± 8 *	73 ± 15 *
HGs RIPC	4 ± 1	4 ± 1	75 ± 5 *^§^	66 ± 3 *
HGl Con	2 ± 1	3 ± 4	100 ± 13 *	93 ± 14 *
HGl RIPC	2 ± 1	2 ± 1	104 ± 16 *	89 ± 14 *
Coronary Flow (mL/min)				
Con	14 ± 2	14 ± 2	6 ± 1 *	6 ± 1 *
RIPC	13 ± 1	13 ± 2	6 ± 3 *	6 ± 2 *
HGs Con	15 ± 2	15 ± 2	8 ± 2 *	7 ± 2 *
HGs RIPC	16 ± 3	15 ± 2	7 ± 1 *	7 ± 1 *
HGl Con	16 ± 1	17 ± 3	5 ± 2 *	5 ± 2 *
HGl RIPC	15 ± 2	15 ± 2	5 ± 1 *	6 ± 1 *
Glucose Levels (mg/dL)				
Con	196 ± 1	200 ± 6	193 ± 3	196 ± 3
RIPC	196 ± 3	200 ± 3	198 ± 6	199 ± 4
HGs Con	194 ± 5	419 ± 27 *	196 ± 3 ^#^	196 ± 3 ^#^
HGs RIPC	196 ± 3	408 ± 23 *	197 ± 2 ^#^	197 ± 2 ^#^
HGl Con	195 ± 2	358 ± 19 *	382 ± 40 *	396 ± 54 *
HGl RIPC	195 ± 4	358 ± 4 *	367 ± 29 *	356 ± 23 *

Data are mean ± SD; Con = control; RIPC = remote ischemic preconditioning; HGs = hyperglycemia short; HGl = hyperglycemia long, * *p* < 0.05 versus baseline; ^#^ *p* < 0.05 versus HGs PC; ^§^ *p* < 0.05.

**Table 3:** Demographic data.

	*n*	Age (Years)	Height (cm)	Body Weight (kg)	BMI (kg/m^2^)
Male	10	25 ± 3	184 ± 5	84 ± 12	24.6 ± 3.4

Data are mean ± SD. BMI = body mass index.

**Table 4:** Criteria for inclusion and exclusion.

Criteria for inclusion	- written informed consent - age: 18–30 years - adequate age-based response to physical stress - normal performance of upper limbs
Criteria for exclusion	- missing consent - long-term medication - acute medication (within the past 14 days) - peripheral arterial disease - diabetes mellitus - hypertension - pre-existing nerve damage of the upper limb - status after thrombo-embolic events - smoking (within the past 5 years, >10 pack years) - chronic pain disorders and psychiatric or neurologic disorders leading to missing legal competence

## Data Availability

Not applicable.
